# Design, synthesis and evaluation of carbazole derivatives as potential antimicrobial agents

**DOI:** 10.1080/14756366.2020.1850713

**Published:** 2021-01-06

**Authors:** Yi-Jie Xue, Ming-Yue Li, Xue-Jun Jin, Chang-Ji Zheng, Hu-Ri Piao

**Affiliations:** a Key Laboratory of Natural Medicines of the Changbai Mountain, Ministry of Education, Yanbian University, Yanji, China;; b School of Pharmacy, Fudan University, Shanghai, China

**Keywords:** Carbazole, antibacterial activities, antifungal activities, structure–activity relationship, cytotoxicity

## Abstract

Five series of novel carbazole derivatives containing an aminoguanidine, dihydrotriazine, thiosemicarbazide, semicarbazide or isonicotinic moiety were designed, synthesised and evaluated for their antimicrobial activities. Most of the compounds exhibited potent inhibitory activities towards different bacterial strains (including one multidrug-resistant clinical isolate) and one fungal strain with minimum inhibitory concentrations (MICs) between 0.5 and 16 µg/ml. Compounds **8f** and **9d** showed the most potent inhibitory activities (MICs of 0.5–2 µg/ml). Furthermore, compounds **8b**, **8d**, **8f**, **8k**, **9b** and **9e** with antimicrobial activities were not cytotoxic to human gastric cancer cell lines (SGC-7901 and AGS) or a normal human liver cell line (L-02). Structure–activity relationship analyses and docking studies implicated the dihydrotriazine group in increasing the antimicrobial potency and reducing the toxicity of the carbazole compounds. *In vitro* enzyme activity assays suggested that compound **8f** binding to dihydrofolate reductase might account for the antimicrobial effect.

## 1. Introduction

The steady increase of microbial pathogens that do not respond to conventional treatments presents a significant threat to global public health. Fungal and bacterial infections are becoming progressively more resistant towards currently available antimicrobial medicines, such as antibiotics. Therefore, there is an urgent requirement for new drugs that target these pathogens. Without effective antimicrobials for prevention and treatment of infections, medical procedures such as organ transplantation, chemotherapy, diabetes management and routine surgery (e.g. caesarean sections or hip replacements) have a significantly higher risk of morbidity and mortality^1^. The scale of the threat of antimicrobial resistance has led to the development of numerous strategies to conserve and make more effective use of existing antibiotics and to promote the development of novel antimicrobials^2^. In 2010, the Infectious Diseases Society of America (IDSA) launched the 10 × 20 initiative that called for the development of 10 novel, safe and effective systemic antibiotics by 2020^3^.

Carbazole alkaloids have attracted considerable attention in medicinal chemistry because they exhibit a broad spectrum of biological and pharmacological activities, including antibacterial, antituberculous, antitumour, antioxidant and anti-inflammatory properties^4–13^. In our previous studies, guanidines exhibited significant antibacterial activity and triazine ring-containing compounds displayed hypoglycaemic, antitumour and antibacterial activities^14^^,^^15^. Dihydrotriazine compounds have a higher hydrophobicity compared with guanidine compounds and several molecules containing this scaffold have been reported as antibacterial agents^16–18^. Recent studies have identified dihydrotriazine-containing compounds as inhibitors of dihydrofolate reductase (DHFR), a ubiquitous enzyme that catalyses the conversion of 7,8-dihydrofolate to 5,6,7,8-tetrahydrofolate, which is involved in metabolic reactions such as purine and thymidine nucleotide biosynthesis^19^^,^^20^. DHFR is required by all organisms to grow and multiply; however, selective inhibitors of microbial enzymes have been utilised as therapeutic agents.

**Figure 1.**
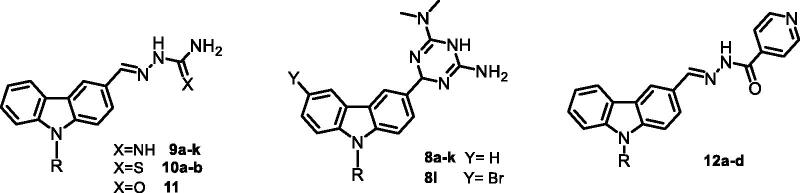
The structures of the target compounds.

Here, we report the design and synthesis of five series of novel carbazole derivatives (Figure 1), totaling 30 compounds and the subsequent *in vitro* evaluations of their antibacterial and antifungal activities. Several different substituents were systematically introduced onto the carbazole ring and their effects on the overall antimicrobial activity were investigated.

## 2. Experimental

### 2.1. Chemistry

All reagents were obtained commercially and were used without further purification. Solvents were dried according to standard procedures. Reactions were monitored by thin-layer chromatography (TLC) on silica gel plates. Melting points were determined in open capillary tubes and were uncorrected. ^1^H NMR and ^13^C NMR spectra were measured on an AV-300 (Bruker, Switzerland), and all chemical shifts were given in ppm relative to TMS. Mass spectra were measured on a HP1100LC (Agilent Technologies). High-resolution mass spectra were measured on an MALDI-TOF/TOF mass spectrometer (Bruker Daltonik, Germany).

#### 2.1.1. Procedure to synthesise compound 2a

To a solution of carbazole (1.03 g, 6.16 mmol) in DMC (10 ml), DABCO (0.069 g, 0.62 mmol) was added and the resulting solution was heated to 90–95 °C for 24 h to give compound **2a**^21^.

#### 2.1.2. Procedure to synthesise compound 2 b

A solution of potassium hydroxide (1.21 g, 21.56 mmol) and bromoethane (1.01 g, 9.24 mmol) in acetone (20 ml) was added to carbazole (1.03 g, 6.16 mmol) and the mixture was stirred at room temperature for 2 h to give compound **2b**^22^.

#### 2.1.3. General procedure to synthesise compounds 2c–k

To a solution of carbazole (1.67 g, 9.00 mmol) in DMF (20 ml) followed by addition of NaH (400 mg, 16.50 mmol) in small portions at 0 °C, over 5 min, benzyl halide (9.90 mmol) was added drop wise at 0 °C, over 5 min, the mixture was stirred at room temperature for 8–18 h to give compounds **2c–k**^23^.

#### 2.1.4. General procedure to synthesise compounds 4a–l

In a three-necked round-bottomed flask, POCl_3_ (700 mg, 4.56 mmol) was added dropwise to DMF (400 mg, 5.42 mmol) while stirring at 0 °C. The mixture was then stirred at room temperature for 1 h. A solution of 9-substituted carbazole (1.93 mmol) was slowly added to this mixture over 5 min at 45 °C, and then the temperature was raised to 95 °C for 8–18 h to yield compounds **4a**–**l**^23^.

#### 2.1.5. General procedure to synthesise compounds 8a–l

A mixture of the required compound **4** (2 mmol) and metformin hydrochloride (2 mmol) in glacial acetic acid (20 ml) was heated under reflux at 120 °C for 4–8 h (completion of the reaction was monitored by TLC). The solvent was then removed under reduced pressure. The crude products were purified by silica gel column chromatography using dichloromethane:methanol (20:1) as the eluent.

##### 2.1.5.1. N^2^,N^2^-Dimethyl-6-(9-methyl-9H-carbazol-3-yl)-3,6-dihydro-1,3,5-triazine-2,4-diamine (8a)

White powder; yield 52.3%; m.p. 267.3–268.9 °C. IR (KBr) cm^−1^: 3300, 3134 (NH_2_, NH), 1606 (C = N). ^1^H NMR (300 MHz, DMSO-*d*_6_, ppm) *δ* 8.88 (br s, 2H, NH_2_), 8.26–8.11 (m, 2H, Ar-H and NH), 7.76–7.46 (m, 5H, Ar-H), 7.25 (t, 1H, *J* = 7.6 Hz, Ar-H), 5.99 (s, 1H, CH), 3.90 (s, 3H, CH_3_), 3.08 (s, 6H, CH_3_). ^13 ^C NMR (75 MHz, DMSO-*d*_6_, ppm) *δ* 157.59, 155.85, 141.05, 140.75, 131.06, 126.01, 123.96, 121.75, 121.63, 120.13, 119.01, 118.02, 109.39 (2 C), 62.77, 36.93 (2 C), 29.12. HRMS (MALDI) calcd for C_18_H_20_N_6_ [M + H]^+^: 321.1822, found: 321.1806.

##### 2.1.5.2. 6-(9-Ethyl-9H-carbazol-3-yl)-N^2^,N^2^-dimethyl-3,6-dihydro-1,3,5-triazine-2,4-diamine (8 b)

White powder; yield 61.6%; m.p. 256.9–259.8 °C. IR (KBr) cm^−1^: 3356, 3132 (NH_2_, NH), 1598 (C = N). ^1^H NMR (300 MHz, DMSO-*d*_6_, ppm) *δ* 8.80 (br s, 2H, NH_2_), 8.25–8.12 (m, 2H, Ar-H), 7.68 (dd, 2H, *J* = 15.0, 8.4 Hz, Ar-H), 7.51 (ddd, 2H, *J* = 16.2, 8.3, 1.5 Hz, Ar-H), 7.24 (t, 1H, *J* = 7.4 Hz, Ar-H), 5.98 (s, 1H, CH), 5.76 (s, 1H, NH), 4.48 (q, 2H, *J* = 7.1 Hz, CH_2_), 3.08 (s, 6H, CH_3_), 1.31 (t, 3H, *J* = 7.0 Hz, CH_3_). ^13 ^C NMR (75 MHz, DMSO-*d*_6_, ppm) *δ* 157.66, 155.83, 139.95, 139.66, 131.06, 126.02, 123.98, 121.95, 121.82, 120.27, 118.98, 118.21, 109.34 (2 C), 62.76, 37.06, 36.94 (2 C), 13.67. HRMS (MALDI) calcd for C_19_H_22_N_6_ [M + H]^+^: 335.1979, found: 335.1967.

##### 2.1.5.3. 6-(9-Benzyl-9H-carbazol-3-yl)-N^2^,N^2^-dimethyl-3,6-dihydro-1,3,5-triazine-2,4-diamine (8c)

White powder; yield 46.1%; m.p. 237.1–238.8 °C. IR (KBr) cm^−1^: 3440, 3240 (NH_2_, NH), 1606 (C = N). ^1^H NMR (300 MHz, DMSO-*d*_6_, ppm) *δ* 8.83 (br s, 2H, NH_2_), 8.27–8.16 (m, 2H, Ar-H), 7.70 (dd, 2H, *J* = 11.5, 8.4 Hz, Ar-H), 7.55–7.44 (m, 2H, Ar-H), 7.25 (dtd, 4H, *J* = 8.1, 6.5, 4.4 Hz, Ar-H), 7.16 (dd, 2H, *J* = 8.0, 1.7 Hz, Ar-H), 5.97 (s, 1H, CH), 5.76 (s, 1H, NH), 5.70 (s, 2H, CH_2_), 3.07 (s, 6H, CH_3_). ^13 ^C NMR (75 MHz, DMSO-*d*_6_, ppm) *δ* 157.62, 155.85, 140.60, 140.29, 137.65, 131.37, 128.59 (2 C), 127.28 (2 C), 126.71, 126.20, 124.18, 122.01, 121.92, 120.30, 119.35, 118.29, 109.84 (2 C), 62.76, 45.67, 36.96, 33.9. HRMS (MALDI) calcd for C_24_H_24_N_6_ [M + H]^+^: 397.2135, found: 397.2134.

##### 2.5.1.4. N^2^,N^2^-Dimethyl-6-(9-(4-methylbenzyl)-9H-carbazol-3-yl)-3,6-dihydro-1,3,5-triazine-2,4-diamine (8d)

White powder; yield 58.8%; m.p. 237.2–239.8 °C. IR (KBr) cm^−1^: 3350, 3037 (NH_2_, NH), 1609 (C = N). ^1^H NMR (300 MHz, DMSO-*d*_6_, ppm) *δ* 8.88 (s, 1H, NH_2_), 8.82 (s, 1H, NH_2_), 8.32–8.14 (m, 2H, Ar-H), 7.69 (dd, 2H, *J* = 12.8, 8.2 Hz, Ar-H), 7.49 (dd, 2H, *J* = 19.7, 8.4 Hz, Ar-H), 7.25 (t, 1H, *J* = 7.4 Hz, Ar-H), 7.07 (d, 4H, *J* = 4.7 Hz, Ar-H), 5.97 (s, 1H, CH), 5.77 (s, 1H, NH), 5.65 (s, 2H, CH_2_), 3.07 (s, 6H, CH_3_), 2.22 (s, 3H, CH_3_). ^13 ^C NMR (75 MHz, DMSO-*d*_6_, ppm) *δ* 158.06, 156.35, 141.06, 140.77, 136.92, 135.05, 131.69, 129.58 (2 C), 127.20 (2 C), 126.63, 124.63, 122.47, 122.40, 120.75, 119.77, 118.79, 110.33 (2 C), 63.34, 55.44, 45.95, 37.41, 21.09. HRMS (MALDI) calcd for C_25_H_26_N_6_ [M + H]^+^: 411.2292, found: 411.2299.

##### 2.5.1.5. 6-(9-(2-Fluorobenzyl)-9H-carbazol-3-yl)-N^2^,N^2^-dimethyl-3,6-dihydro-1,3,5-triazine-2,4-diamine (8e)

White powder; yield 40.1%; m.p. 201.2–205.0 °C. IR (KBr) cm^−1^: 3423, 3144 (NH_2_, NH), 1603 (C = N). ^1^H NMR (300 MHz, DMSO-*d*_6_, ppm) *δ* 8.90 (s, 1H, NH_2_), 8.84 (s, 1H, NH_2_), 8.30–8.16 (m, 2H, Ar-H), 7.68 (dd, 2H, *J* = 13.2, 8.3 Hz, Ar-H), 7.56–7.43 (m, 2H, Ar-H), 7.33–7.22 (m, 3H, Ar-H), 7.03 (td, 1H, *J* = 7.4, 1.6 Hz, Ar-H), 6.88–6.78 (m, 1H, Ar-H), 5.97 (s, 1H, CH), 5.77 (s, 3H, NH and CH_2_), 3.07 (s, 6H, CH_3_). ^13 ^C NMR (75 MHz, DMSO-*d*_6_, ppm) *δ* 161.69, 158.44, 157.61, 155.96, 140.57, 140.28, 131.43, 129.50 (d, 1 C, *J* = 8.1 Hz), 128.71 (d, 1 C, *J* = 4.2 Hz), 126.23, 124.57 (d, 1 C, *J* = 3.4 Hz), 124.22, 124.04, 122.08 (d, 1 C, *J* = 2.3 Hz), 120.28, 119.50, 118.34, 115.65, 115.37, 109.75, 62.92, 62.01, 56.01, 36.95. HRMS (MALDI) calcd for C_24_H_23_FN_6_ [M + H]^+^: 415.2041, found: 415.2037.

##### 2.5.1.6. 6-(9-(2,4-Dichlorobenzyl)-9H-carbazol-3-yl)-N^2^,N^2^-dimethyl-3,6-dihydro-1,3,5-triazine-2,4-diamine (8f)

White powder; yield 40.1%; m.p. 255.2–257.1 °C. IR (KBr) cm^−1^: 3240, 3060 (NH_2_, NH), 1603 (C = N). ^1^H NMR (300 MHz, DMSO-*d*_6_, ppm) *δ* 8.93–8.72 (br s, 2H, NH_2_), 8.35–8.18 (m, 2H, Ar-H), 7.75 (d, 1H, *J* = 2.1 Hz, Ar-H), 7.68–7.17 (m, 7H, Ar-H), 6.33 (d, 1H, *J* = 8.4 Hz, CH), 5.97 (s, 1H, NH), 5.76 (s, 2H, CH_2_), 3.08 (s, 6H, CH_3_). ^13^C NMR (75 MHz, DMSO-*d*_6_, ppm) *δ* 157.55, 155.97, 140.52, 140.25, 133.76, 132.77, 132.69, 131.74, 129.10, 128.50, 127.69, 126.43, 124.41, 122.17, 120.43 (2 C), 119.76, 118.48, 109.63 (2 C), 62.92, 45.32, 43.47, 36.95. HRMS (MALDI) calcd for C_24_H_22_Cl_2_N_6_ [M + H]^+^: 465.1356, found: 465.1359. Purity: 97.41% by HPLC (A: 0.1% FA in H_2_O; B: 0.1% FA in CH_3_CN, graded: 20–100%), t_R_ 12.273 min, λ: 250 nm.

##### 2.1.5.7. 6-(9-(2-Chlorobenzyl)-9H-carbazol-3-yl)-N^2^,N^2^-dimethyl-3,6-dihydro-1,3,5-triazine-2,4-diamine (8 g)

White powder; yield 37.6%; m.p. 240.7–243.2 °C. IR (KBr) cm^−1^: 3396, 3211 (NH_2_, NH), 1603 (C = N). ^1^H NMR (300 MHz, DMSO-*d*_6_, ppm) *δ* 8.86 (s, 1H, NH_2_), 8.81 (s, 1H, NH_2_), 8.38–8.20 (m, 2H, Ar-H), 7.70–7.39 (m, 6H, Ar-H), 7.37–7.23 (m, 2H, Ar-H), 7.11 (dd, 1H, *J* = 8.8, 6.3 Hz, Ar-H), 6.36 (d, 1H, *J* = 8.0 Hz, CH), 5.98 (s, 1H, NH), 5.84–5.75 (m, 2H, CH_2_), 3.09 (s, 6H, CH_3_). ^13 ^C NMR (75 MHz, DMSO-*d*_6_, ppm) *δ* 157.99, 156.37, 141.07, 140.80, 134.95, 132.24, 132.07, 130.10, 129.52, 127.97, 127.59, 126.86, 124.86, 122.57, 122.54, 120.90, 120.13, 118.95, 110.13 (2 C), 63.36, 55.43, 44.22, 37.42. HRMS (MALDI) calcd for C_24_H_23_ClN_6_ [M + H]^+^: 431.1745, found: 431.1733.

##### 2.1.5.8. 6-(9-(3-Chlorobenzyl)-9H-carbazol-3-yl)-N^2^,N^2^-dimethyl-3,6-dihydro-1,3,5-triazine-2,4-diamine (8 h)

White powder; yield 54.9%; m.p. 207.0–209.2 °C. IR (KBr) cm^−1^: 3313, 3029 (NH_2_, NH), 1600 (C = N). ^1^H NMR (300 MHz, DMSO-*d*_6_, ppm) *δ* 8.87 (s, 1H, NH_2_), 8.81 (s, 1H, NH_2_), 8.34–8.16 (m, 2H, Ar-H), 7.71 (dd, 2H, *J* = 13.6, 8.3 Hz, Ar-H), 7.59–7.45 (m, 2H, Ar-H), 7.39–7.20 (m, 4H, Ar-H), 7.06 (dd, 1H, *J* = 6.3, 2.9 Hz, Ar-H), 5.97 (s, 1H, CH), 5.77 (s, 1H, NH), 5.74 (s, 2H, CH_2_), 3.12 (s, 6H, CH_3_). ^13 ^C NMR (75 MHz, DMSO-*d*_6_, ppm) *δ* 158.06, 156.36, 140.96, 140.74, 140.69, 133.67, 131.94, 131.03, 127.78, 127.03, 126.80, 125.77, 124.82, 122.52, 122.46, 120.85, 120.02, 118.91, 110.23 (2 C), 63.34, 55.44, 45.55, 37.42. HRMS (MALDI) calcd for C_24_H_23_ClN_6_ [M + H]^+^: 431.1745, found: 431.1749.

##### 2.1.5.9. 6-(9-(4-Chlorobenzyl)-9H-carbazol-3-yl)-N^2^,N^2^-dimethyl-3,6-dihydro-1,3,5-triazine-2,4-diamine (8i)

White powder; yield 36.7%; m.p. 262.4–264.1 °C. IR (KBr) cm^−1^: 3373, 3059 (NH_2_, NH), 1603 (C = N). ^1^H NMR (300 MHz, DMSO-*d*_6_, ppm) *δ* 9.00 (s, 1H, NH_2_), 8.94 (s, 1H, NH_2_), 8.36–8.14 (m, 2H, Ar-H), 7.69 (dd, 2H, *J* = 14.6, 8.2 Hz, Ar-H), 7.60–7.43 (m, 3H, Ar-H), 7.35 (dd, 2H, *J* = 8.6, 2.1 Hz, Ar-H), 7.29–7.13 (m, 3H, Ar-H and NH), 5.98 (s, 1H, CH), 5.72 (s, 2H, CH_2_), 3.07 (s, 6H, CH_3_). ^13 ^C NMR (75 MHz, DMSO-*d*_6_, ppm) *δ* 158.02, 156.35, 140.95, 140.67, 137.15, 132.36, 131.92, 129.05 (4 C), 126.75, 124.73, 122.52, 122.46, 120.83, 119.96, 118.85, 110.26 (2 C), 63.32, 49.04, 45.47, 37.41. HRMS (MALDI) calcd for C_24_H_23_ClN_6_ [M + H]^+^: 431.1745, found: 431.1761.

##### 2.1.5.10. 6-(9-(4-Bromobenzyl)-9H-carbazol-3-yl)-N^2^,N^2^-dimethyl-3,6-dihydro-1,3,5-triazine-2,4-diamine (8j)

White powder; yield 35.1%; m.p. 248.7–252.1 °C. IR (KBr) cm^−1^: 3311, 3054 (NH_2_, NH), 1603 (C = N). ^1^H NMR (300 MHz, DMSO-*d*_6_, ppm) *δ* 9.06 (s, 1H, NH_2_), 8.99 (s, 1H, NH_2_), 8.34–8.13 (m, 2H, Ar-H), 7.68 (dd, 2H, *J* = 15.2, 8.3 Hz, Ar-H), 7.63–7.41 (m, 5H, Ar-H), 7.25 (t, 1H, *J* = 7.5 Hz, Ar-H), 7.17–7.06 (m, 2H, Ar-H and NH), 5.99 (s, 1H, CH), 5.69 (s, 2H, CH_2_), 3.07 (s, 6H, CH_3_). ^13 ^C NMR (75 MHz, DMSO-*d*_6_, ppm) *δ* 157.53, 155.87, 140.47, 140.20, 137.10, 131.49(4 C), 128.91 (2 C), 126.28, 124.27, 122.04, 121.98, 120.37, 119.49, 118.39, 109.79 (2 C), 62.86, 45.04, 36.94 (2 C). HRMS (MALDI) calcd for C_24_H_23_BrN_6_ [M + H]^+^: 475.1240, found: 475.1230.

##### 2.1.5.11. 4-((3-(4-Amino-6-(dimethylamino)-2,5-dihydro-1,3,5-triazin-2-yl)-9H-carbazol-9-yl)methyl)benzonitrile (8k)

White powder; yield 52.9%; m.p. 234.1–235.4 °C. IR (KBr) cm^−1^: 3306, 3061 (NH_2_, NH), 1605 (C = N). ^1^H NMR (300 MHz, DMSO-*d*_6_, ppm) *δ* 9.06 (s, 1H, NH_2_), 9.01 (s, 1H, NH_2_), 8.35–8.15 (m, 2H, Ar-H), 7.78–7.62 (m, 4H, Ar-H), 7.56 (d, 1H, *J* = 8.4 Hz, Ar-H), 7.51–7.43 (m, 1H, Ar-H), 7.36–7.23 (m, 3H, Ar-H), 6.01 (s, 1H, CH), 5.83 (s, 2H, CH_2_), 5.77 (s, 1H, NH), 3.09 (s, 6H, CH_3_). ^13 ^C NMR (75 MHz, DMSO-*d*_6_, ppm) *δ* 158.06, 156.33, 143.92, 140.95, 140.66, 133.04 (2 C), 132.15, 127.98 (2 C), 126.82, 124.80, 122.58, 122.54, 120.87, 120.10, 119.11, 118.88, 110.59, 110.18 (2 C), 63.28, 55.41, 49.03, 37.42. HRMS (MALDI) calcd for C_25_H_23_N_7_ [M + H]^+^: 422.2088, found: 422.2077.

##### 2.1.5.12. 6-(6-Bromo-9-phenyl-9H-carbazol-3-yl)-N^2^,N^2^-dimethyl-3,6-dihydro-1,3,5-triazine-2,4-diamine (8 l)

White powder; yield 41.4%; m.p. 221.5–224.8 °C. IR (KBr) cm^−1^: 3383, 3070 (NH_2_, NH), 1600 (C = N). ^1^H NMR (300 MHz, DMSO-*d*_6_, ppm) *δ* 9.19 (s, 2H, NH_2_), 8.44 (d, 2H, *J* = 19.4 Hz, Ar-H), 7.64 (dd, 4H, *J* = 15.7, 8.1 Hz, Ar-H), 7.55 (d, 3H, *J* = 7.6 Hz, Ar-H), 7.39 (d, 1H, *J* = 8.4 Hz, Ar-H), 7.29 (t, 1H, *J* = 6.9 Hz, Ar-H), 6.05 (s, 1H, CH), 5.76 (s, 1H, NH), 3.08 (s, 6H, CH_3_). ^13 ^C NMR (75 MHz, DMSO-*d*_6_, ppm) *δ* 158.01, 156.25, 141.08, 139.79, 136.62, 133.51, 130.71 (2 C), 129.41, 128.52, 127.02 (2 C), 125.85, 124.88, 123.47, 121.88, 119.35, 112.82, 112.18, 110.60, 63.04, 55.41, 49.03. HRMS (MALDI) calcd for C_23_H_21_BrN_6_ [M + H]^+^: 461.1084, found: 461.1066. Purity: 98.69% by HPLC (A: 0.1% FA in H_2_O; B: 0.1% FA in CH_3_CN, graded: 20–100%), t_R_ 11.660 min, λ: 250 nm.

#### 2.1.6. General procedure to synthesise compounds 9a–k

A mixture of the required compound **4** (2.3 mmol) and aminoguanidine hydrochloride (2 mmol) in ethanol (20 ml) in the presence of 5 drops of concentrated hydrochloric acid was stirred at 50–60 °C for 8–12 h. The solvent was then removed under reduced pressure. The crude products were purified by silica gel column chromatography using dichloromethane:methanol (10:1) as the eluent.

##### 2.1.6.1. (E)-2-((9-Methyl-9H-carbazol-3-yl)methylene)hydrazine-1-carboximidamide (9a)

White powder; yield 52.3%; m.p. 199.1-200.8 °C. IR (KBr) cm^−1^: 3330, 3147 (NH_2_, NH), 1629 (C = N). ^1^H NMR (300 MHz, DMSO-*d*_6_, ppm) *δ* 11.89 (br s, 1H, NH), 8.63 (s, 1H, CH = N), 8.33 (s, 1H, Ar-H), 8.18 (d, 1H, *J* = 8.0 Hz, Ar-H), 8.05 (d, 1H, *J* = 8.4 Hz, Ar-H), 7.91–7.46 (m, 6H, Ar-H and NH), 7.29 (d, 1H, *J* = 7.5 Hz, Ar-H), 3.94 (s, 3H, CH_3_). ^13 ^C NMR (75 MHz, DMSO-*d*_6_, ppm) *δ* 155.41, 147.77, 141.81, 141.07, 126.20, 125.14, 124.36, 122.08, 121.96, 120.59, 120.36, 119.41, 109.53, 109.40, 29.16. HRMS (MALDI) calcd for C_15_H_15_N_5_ [M + H]^+^: 266.1400, found: 266.1405.

##### 2.1.6.2. (E)-2-((9-Ethyl-9H-carbazol-3-yl)methylene)hydrazine-1-carboximidamide (9b)

White powder; yield 59.2%; m.p. 199.4–200.5 °C. IR (KBr) cm^−1^: 3354, 3120 (NH_2_, NH), 1628 (C = N). ^1^H NMR (300 MHz, DMSO-*d*_6_, ppm) *δ* 11.85 (br s, 1H, NH), 8.63 (s, 1H, CH = N), 8.33 (d, 1H, *J* = 1.9 Hz, Ar-H), 8.18 (d, 1H, *J* = 7.7 Hz, Ar-H), 8.03 (d, 1H, *J* = 8.6 Hz, Ar-H), 7.77–7.46 (m, 6H, Ar-H and NH), 7.27 (t, 1H, *J* = 7.3 Hz, Ar-H), 4.49 (q, 2H, *J* = 6.9 Hz, CH_2_), 1.33 (t, 3H, *J* = 7.1 Hz, CH_3_). ^13 ^C NMR (75 MHz, DMSO-*d*_6_, ppm) *δ* 155.42, 147.76, 140.83, 140.04, 131.54, 128.68, 126.26, 125.23, 124.44, 122.31, 120.72, 120.53, 119.42, 109.56, 109.40, 37.18, 18.68. HRMS (MALDI) calcd for C_16_H_17_N_5_ [M + H]^+^: 280.1557, found: 280.1548.

##### 2.1.6.3. (E)-2-((9-Benzyl-9H-carbazol-3-yl)methylene)hydrazine-1-carboximidamide (9c)

White powder; yield 63.1%; m.p. 271.0–272.4 °C. IR (KBr) cm^−1^: 3319, 3154 (NH_2_, NH), 1600 (C = N). ^1^H NMR (300 MHz, DMSO-*d*_6_, ppm) *δ* 11.91 (br s, 1H, NH), 8.67 (s, 1H, CH = N), 8.34 (s, 1H, Ar-H), 8.21 (d, 1H, *J* = 7.7 Hz, Ar-H), 8.01 (d, 1H, *J* = 8.5 Hz, Ar-H), 7.84–7.65 (m, 5H, Ar-H and NH), 7.47 (t, 1H, *J* = 7.7 Hz, Ar-H), 7.24 (dt, 6H, *J* = 18.8, 7.5 Hz, Ar-H), 5.71 (s, 2H, CH_2_). ^13 ^C NMR (75 MHz, DMSO-*d*_6_, ppm) *δ* 155.82, 148.14, 141.94, 141.09, 137.95, 132.14, 131.97, 129.05, 127.80, 127.21, 126.85, 125.85, 125.26, 122.87, 122.73, 121.10, 120.99, 120.21, 110.50, 110.33, 46.20. HRMS (MALDI) calcd for C_21_H_19_N_5_ [M + H]^+^: 342.1713, found: 342.1701.

##### 2.1.6.4. (E)-2-((9-(4-Methylbenzyl)-9H-carbazol-3-yl)methylene)hydrazine-1-carboximidamide (9d)

White powder; yield 45.0%; m.p. 295.5–298.0 °C. IR (KBr) cm^−1^: 3382, 3134 (NH_2_, NH), 1629 (C = N). ^1^H NMR (300 MHz, DMSO-*d*_6_, ppm) *δ* 11.82 (br s, 1H, NH), 8.59 (s, 1H, CH = N), 8.25 (s, 1H, Ar-H), 8.13 (d, 1H, *J* = 7.7 Hz, Ar-H), 7.94 (dd, 1H, *J* = 8.7, 1.6 Hz, Ar-H), 7.75–7.58 (m, 4H, Ar-H and NH), 7.40 (t, 2H, *J* = 7.7 Hz, Ar-H), 7.20 (t, 1H, *J* = 7.4 Hz, Ar-H), 7.01 (s, 4H, Ar-H), 5.59 (s, 2H, CH_2_), 2.14 (s, 3H, CH_3_). ^13 ^C NMR (75 MHz, DMSO-*d*_6_, ppm) *δ* 155.46, 147.63, 141.47, 140.63, 136.53, 134.46, 129.14 (2 C), 126.81 (2 C), 126.37, 125.39, 124.78, 122.43, 122.30, 120.65, 120.54, 119.71, 110.09, 109.91, 45.56, 20.63. HRMS (MALDI) calcd for C_22_H_21_N_5_ [M + H]^+^: 356.1869, found: 356.1854. Purity: 96.74% by HPLC (A: 0.1% FA in H_2_O; B: 0.1% FA in CH_3_CN, graded: 20–100%), t_R_ 11.533 min, λ: 250 nm.

##### 2.1.6.5. (E)-2-((9-(2-Fluorobenzyl)-9H-carbazol-3-yl)methylene)hydrazine-1-carboximidamide (9e)

White powder; yield 39.3%; m.p. 248.3–249.9 °C. IR (KBr) cm^−1^: 3356, 3163 (NH_2_, NH), 1637 (C = N). ^1^H NMR (300 MHz, DMSO-*d*_6_, ppm) *δ* 11.88 (s, 1H, NH_2_), 8.67 (s, 1H, CH = N), 8.32 (s, 1H, Ar-H), 8.21 (d, 1H, *J* = 7.8 Hz, Ar-H), 8.02 (dd, 1H, *J* = 8.7, 1.6 Hz, Ar-H), 7.89–7.41 (m, 6H, Ar-H and NH), 7.37–7.19 (m, 3H, Ar-H), 7.04 (td, 1H, *J* = 7.4, 1.4 Hz, Ar-H), 6.89 (td, 1H, *J* = 7.7, 1.7 Hz, Ar-H), 5.77 (d, 2H, *J* = 2.2 Hz, CH_2_). ^13 ^C NMR (75 MHz, DMSO-*d*_6_, ppm) *δ* 161.68, 158.43, 155.40, 147.52, 141.38, 140.51, 128.75 (d, 1 C, *J* = 4.1 Hz), 126.40, 125.36, 124.93, 124.57 (d, 1 C, *J* = 3.3 Hz), 124.12, 123.92, 122.43, 122.30, 120.58 (d, 1 C, *J* = 8.3 Hz), 119.85, 115.67, 115.39, 109.89 (d, 1 C, *J* = 8.6 Hz), 65.01. HRMS (MALDI) calcd for C_21_H_18_FN_5_ [M + H]^+^: 360.1619, found: 360.1611.

##### 2.1.6.6. (E)-2-((9-(2,4-Dichlorobenzyl)-9H-carbazol-3-yl)methylene)hydrazine-1-carboximidamide (9f)

White powder; yield 58.2%; m.p. 240.7–242.8 °C. IR (KBr) cm^−1^: 3397, 3146 (NH_2_, NH), 1629 (C = N). ^1^H NMR (300 MHz, DMSO-*d*_6_, ppm) *δ* 12.12 (br s, 1H, NH), 8.71 (s, 1H, CH = N), 8.35 (s, 1H, Ar-H), 8.24 (d, 1H, *J* = 7.7 Hz, Ar-H), 8.11–7.66 (m, 5H, Ar-H and NH), 7.59 (d, 1H, *J* = 8.6 Hz, Ar-H), 7.54–7.41 (m, 2H, Ar-H), 7.29 (t, 1H, *J* = 7.3 Hz, Ar-H), 7.19 (dd, 1H, *J* = 8.3, 2.3 Hz, Ar-H), 6.41 (d, 1H, *J* = 8.4 Hz, Ar-H), 5.75 (s, 2H, CH_2_). ^13 ^C NMR (75 MHz, DMSO-*d*_6_, ppm) *δ* 155.52, 147.63, 141.45, 140.55, 133.66, 132.83, 132.76, 129.13, 128.66, 127.72, 126.61, 125.56, 125.24, 122.63, 122.46, 120.74, 120.68, 120.12, 109.83, 109.73, 43.59. HRMS (MALDI) calcd for C_21_H_17_Cl_2_N_5_ [M + H]^+^: 410.0934, found: 410.0932.

##### 2.1.6.7. (E)-2-((9-(2-Chlorobenzyl)-9H-carbazol-3-yl)methylene)hydrazine-1-carboximidamide (9 g)

White powder; yield 45.4%; m.p. 249.7–250.4 °C. IR (KBr) cm^−1^: 3386, 3159 (NH_2_, NH), 1629 (C = N). ^1^H NMR (300 MHz, DMSO-*d*_6_, ppm) *δ* 11.88 (br s, 1H, NH), 8.70 (s, 1H, CH = N), 8.33 (s, 1H, Ar-H), 8.24 (d, 1H, *J* = 7.6 Hz, Ar-H), 8.00 (dd, *J* = 8.6, 1H, 1.7 Hz, Ar-H), 7.96–7.43 (m, 7H, Ar-H and NH), 7.37–7.24 (m, 2H, Ar-H), 7.18–7.06 (m, 1H, Ar-H), 6.51–6.40 (m, 1H, Ar-H), 5.77 (s, 2H, CH_2_). ^13 ^C NMR (75 MHz, DMSO-*d*_6_, ppm) *δ* 155.88, 147.98, 141.96, 141.05, 134.82, 132.26, 130.10, 129.55, 127.95, 127.71, 126.99, 125.94, 125.55, 122.97, 122.83, 121.20, 121.10, 120.45, 110.29, 110.18, 44.30. HRMS (MALDI) calcd for C_21_H_18_ClN_5_ [M + H]^+^: 376.1326, found: 376.1322.

##### 2.1.6.8. (E)-2-((9-(3-Chlorobenzyl)-9H-carbazol-3-yl)methylene)hydrazine-1-carboximidamide (9 h)

White powder; yield 41.5%; m.p. 256.1–257.5 °C. IR (KBr) cm^−1^: 3389, 3137 (NH_2_, NH), 1634 (C = N). ^1^H NMR (300 MHz, DMSO-*d*_6_, ppm) *δ* 11.93 (s, 1H, NH), 8.68 (s, 1H, CH = N), 8.33 (s, 1H, Ar-H), 8.22 (d, 1H, *J* = 7.7 Hz, Ar-H), 8.09–7.58 (m, 6H, Ar-H and NH), 7.49 (t, 1H, *J* = 7.7 Hz, Ar-H), 7.29 (t, 4H, *J* = 6.0 Hz, Ar-H), 7.08 (dd, 1H, *J* = 6.6, 3.1 Hz, Ar-H), 5.75 (s, 2H, CH_2_). ^13 ^C NMR (75 MHz, DMSO-*d*_6_, ppm) *δ* 155.89, 148.00, 141.82, 140.97, 140.57, 133.66, 131.02, 127.81, 127.07, 126.96, 125.94, 125.83, 125.46, 122.91, 122.77, 121.19, 121.07, 120.38, 110.43, 110.28, 45.59. HRMS (MALDI) calcd for C_21_H_18_ClN_5_ [M + H]^+^: 376.1326, found: 376.1319.

##### 2.1.6.9. (E)-2-((9-(4-Chlorobenzyl)-9H-carbazol-3-yl)methylene)hydrazine-1-carboximidamide (9i)

White powder; yield 38.7%; m.p. 239.5–240.8 °C. IR (KBr) cm^−1^: 3266, 3057 (NH_2_, NH), 1629 (C = N). ^1^H NMR (300 MHz, DMSO-*d*_6_, ppm) *δ* 12.07 (br s, 1H, NH), 8.68 (s, 1H, CH = N), 8.35 (d, 1H, *J* = 5.4 Hz, Ar-H), 8.21 (d, 1H, *J* = 7.7 Hz, Ar-H), 8.01 (d, 1H, *J* = 8.3 Hz, Ar-H), 7.68 (m, 5H, Ar-H and NH), 7.47 (t, 1H, *J* = 7.7 Hz, Ar-H), 7.38–7.24 (m, 3H, Ar-H), 7.18 (d, 2H, *J* = 8.7 Hz, Ar-H), 5.72 (s, 2H, CH_2_). ^13 ^C NMR (75 MHz, DMSO-*d*_6_, ppm) *δ* 155.44, 147.58, 141.38, 140.53, 136.56, 131.96, 128.66 (2 C), 128.62 (2 C), 126.48, 125.47, 124.96, 122.49, 122.35, 120.72, 120.62, 119.88, 110.02, 109.86, 45.08. HRMS (MALDI) calcd for C_21_H_18_ClN_5_ [M + H]^+^: 376.1326, found: 376.1329.

##### 2.1.6.10. (E)-2-((9-(4-Bromobenzyl)-9H-carbazol-3-yl)methylene)hydrazine-1-carboximidamide (9j)

White powder; yield 41.1%; m.p. 239.5–240.7 °C. IR (KBr) cm^−1^: 3377, 3161 (NH_2_, NH), 1629 (C = N). ^1^H NMR (300 MHz, DMSO-*d*_6_, ppm) *δ* 11.86 (s, 1H, NH), 8.67 (s, 1H, CH = N), 8.32 (s, 1H, Ar-H), 8.21 (d, 1H, *J* = 7.7 Hz, Ar-H), 8.09–7.98 (m, 1H, Ar-H), 7.97–7.56 (m, 5H, Ar-H and NH), 7.48 (dt, 3H, *J* = 7.0, 3.5 Hz, Ar-H), 7.29 (t, 1H, *J* = 7.5 Hz, Ar-H), 7.13 (d, 2H, *J* = 8.5 Hz, Ar-H), 5.72 (s, 2H, CH_2_). ^13 ^C NMR (75 MHz, DMSO-*d*_6_, ppm) *δ* 155.43, 147.62, 141.39, 140.54, 136.98, 131.54 (2 C), 129.00 (2 C), 126.49, 125.48, 124.96, 122.49, 122.35, 120.71, 120.61, 120.46, 119.89, 110.02, 109.85, 45.16. HRMS (MALDI) calcd for C_21_H_18_BrN_5_ [M + H]^+^: 420.0818, found: 420.0829.

##### 2.1.6.11. (E)-2-((9-(4-Cyanobenzyl)-9H-carbazol-3-yl)methylene)hydrazine-1-carboximidamide (9k)

White powder; yield 45.7%; m.p. 240.6–243.9 °C. IR (KBr) cm^−1^: 3300, 3123 (NH_2_, NH), 1631 (C = N). ^1^H NMR (300 MHz, DMSO-*d*_6_, ppm) *δ* 11.92 (br s, 1H, NH), 8.68 (s, 1H, CH = N), 8.33 (s, 1H, Ar-H), 8.22 (d, 1H, *J* = 7.7 Hz, Ar-H), 8.17–7.55 (m, 8H, Ar-H and NH), 7.48 (t, 1H, *J* = 7.7 Hz, Ar-H), 7.30 (dd, 3H, *J* = 7.9, 2.2 Hz, Ar-H), 5.85 (s, 2H, CH_2_). ^13 ^C NMR (75 MHz, DMSO-*d*_6_, ppm) *δ* 155.31, 147.68, 143.30, 141.37, 140.50, 132.61 (2 C), 127.56 (2 C), 126.60, 125.57, 125.09, 122.54, 122.35, 120.71 (2 C), 120.05, 118.65, 110.15, 109.91, 109.79, 45.43. HRMS (MALDI) calcd for C_22_H_18_N_6_ [M + H]^+^: 367.1666, found: 367.1656.

#### 2.1.7. General procedure to synthesise compounds 10a–b and 11

A mixture of the required compound **4** (2 mmol) and either thiosemicarbazide hydrochloride or semicarbazide hydrochloride(2 mmol) in ethanol (20 ml) was stirred at 50–60 °C for 8–12 h in the presence of five drops of concentrated hydrochloric acid. The solution was evaporated to dryness under reduced pressure, and the residue was purified by silica gel column chromatography using dichloromethane:methanol (80:1) as the eluent.

##### 2.1.7.1. (E)-2-((9-Methyl-9H-carbazol-3-yl)methylene)hydrazine-1-carbothioamide (10a)

White solid; yield 60.5%; m.p. 222.4–223.8 °C. IR (KBr) cm^−1^: 3238, 3026 (NH_2_, NH), 1630 (C = N), 1120 (C = S). ^1^H NMR (300 MHz, DMSO-*d*_6_, ppm) *δ* 11.42 (s, 1H, NH), 8.59 (s, 1H, CH = N), 8.21 (d, 3H, *J* = 7.8 Hz, Ar-H), 8.08–7.89 (m, 2H, NH_2_), 7.62 (d, 2H, *J* = 8.6 Hz, Ar-H), 7.50 (t, 1H, *J* = 7.6 Hz, Ar-H), 7.25 (t, 1H, *J* = 7.4 Hz, Ar-H), 3.90 (s, 3H, CH_3_). ^13 ^C NMR (75 MHz, DMSO-*d*_6_, ppm) *δ* 177.45, 143.65, 141.54, 141.02, 126.11, 125.11, 125.02, 122.19, 122.00, 120.47, 120.19, 119.30, 109.41, 109.36, 29.09. HRMS (MALDI) calcd for C_15_H_14_N_4_S [M + H]^+^: 283.1012, found: 283.1020.

##### 2.1.7.2. (E)-2-((9-Ethyl-9H-carbazol-3-yl)methylene)hydrazine-1-carbothioamide (10 b)

White solid; yield 64.6%; m.p. 199.8–202.0 °C. IR (KBr) cm^−1^: 3280, 3043 (NH_2_, NH), 1594 (C = N), 1200 (C = S). ^1^H NMR (300 MHz, DMSO-*d*_6_, ppm) *δ* 11.40 (s, 1H, NH), 8.58 (s, 1H, CH = N), 8.27–8.13 (m, 3H, Ar-H), 8.04–7.91 (m, 2H, Ar-H), 7.64 (s, 1H, NH_2_), 7.61 (s, 1H, NH_2_), 7.53–7.44 (m, 1H, Ar-H), 7.24 (t, 1H, *J* = 7.4 Hz, Ar-H), 4.45 (q, 2H, *J* = 6.9 Hz, CH_2_), 1.32 (t, 3H, *J* = 6.8 Hz, CH_3_). ^13 ^C NMR (75 MHz, DMSO-*d*_6_, ppm) *δ* 177.45, 143.56, 140.52, 139.96, 126.12, 125.13, 125.08, 122.39, 122.21, 120.61, 120.24, 119.25, 109.39, 109.29, 37.10, 18.64. HRMS (MALDI) calcd for C_16_H_16_N_4_S [M + H]^+^: 297.1168, found: 297.1161.

##### 2.1.7.3. (E)-2-((9-Ethyl-9H-carbazol-3-yl)methylene)hydrazine-1-carboxamide (11)

White solid; yield 42.3%; m.p. 211.1–212.0 °C. IR (KBr) cm^−1^: 3343, 3049 (NH_2_, NH), 1691 (C = O), 1595 (C = N). ^1^H NMR (300 MHz, DMSO-*d*_6_, ppm) *δ* 10.17 (s, 1H, NH), 8.51 (s, 1H, CH = N), 8.20 (d, 1H, *J* = 7.7 Hz, Ar-H), 8.01 (s, 1H, Ar-H), 7.86 (d, 1H, *J* = 8.5 Hz, Ar-H), 7.61 (dd, 2H, *J* = 8.4, 3.7 Hz, Ar-H), 7.47 (t, 1H, *J* = 7.7 Hz, Ar-H), 7.23 (t, 1H, *J* = 7.4 Hz, Ar-H), 6.52 (br s, 2H, NH_2_), 4.46 (q, 2H, *J* = 7.0 Hz, CH_2_), 1.32 (t, 3H, *J* = 6.9 Hz, CH_3_). ^13 ^C NMR (75 MHz, DMSO-*d*_6_, ppm) *δ* 157.03, 140.48, 140.05, 139.93, 126.01, 125.89, 124.46, 122.33, 122.22, 120.58, 119.22, 119.08, 109.31, 109.19, 37.06, 13.72. HRMS (MALDI) calcd for C_16_H_16_N_4_O [M + H]^+^: 281.1397, found: 281.1392.

#### 2.1.8. General procedure for the synthesis of compounds 12a–12d

The intermediate **4** (2 mmol) reacted with isonicotinic acid hydrazide (2 mmol) in the presence of catalytic amounts of hydrochloric acid (5 drops) in ethanol (20 ml) at 70 °C for 5 h. The solution was evaporated to dryness under reduced pressure, and the residue was purified by silica gel column chromatography with (dichloromethane: methanol = 60:1).

##### 2.1.8.1. (E)-N'-((9-Benzyl-9H-carbazol-3-yl)methylene)isonicotinohydrazide (12a)

Yellow solid; yield 80.0%; m.p. 198.0–199.5 °C. IR (KBr) cm^−1^: 3250, 3046 (NH), 1646 (C = O), 1601 (C = N). ^1^H NMR (300 MHz, DMSO-*d*_6_, ppm) *δ* 12.02 (s, 1H, NH), 8.90–8.73 (m, 2H, Ar-H), 8.65 (s, 1H, CH = N), 8.55 (s, 1H, Ar-H), 8.31 (dd, 1H, *J* = 9.6, 6.5 Hz, Ar-H), 7.97–7.63 (m, 5H, Ar-H and pyridine-H), 7.48 (dd, 1H, *J* = 10.7, 4.7 Hz, Ar-H), 7.38–7.06 (m, 6H, Ar-H), 5.73 (s, 2H, CH_2_). ^13 ^C NMR (75 MHz, DMSO-*d*_6_, ppm) *δ* 161.44, 150.29 (2 C), 141.50, 140.77, 140.70, 137.43, 128.59 (2 C), 127.32 (2 C), 126.70 (2 C), 126.40, 125.41, 125.04, 122.60, 122.25, 121.54 (2 C), 120.69, 120.29, 119.74, 110.03, 109.92, 45.81. HRMS (MALDI) calcd for C_26_H_20_N_4_O [M + H]^+^: 405.1710, found: 405.1719.

##### 2.1.8.2. (E)-N'-((9-(4-Methylbenzyl)-9H-carbazol-3-yl)methylene)isonicotinohydrazide (12 b)

Yellow solid; yield 74.7%; m.p. 196.0–197.8 °C. IR (KBr) cm^−1^: 3320, 3060 (NH), 1647 (C = O), 1595 (C = N). ^1^H NMR (300 MHz, DMSO-*d*_6_, ppm) *δ* 12.02 (s, 1H, NH), 8.80 (d, 2H, *J* = 5.0 Hz, Ar-H), 8.63 (s, 1H, CH = N), 8.54 (s, 1H, pyridine–H), 8.29 (d, 1H, *J* = 7.4 Hz, pyridine–H), 7.95–7.81 (m, 3H, Ar-H), 7.71 (dd, 2H, *J* = 23.9, 8.3 Hz, pyridine–H), 7.48 (t, 1H, *J* = 7.6 Hz, Ar-H), 7.27 (t, 1H, *J* = 7.4 Hz, Ar-H), 7.09 (s, 4H, Ar-H), 5.66 (s, 2H, CH_2_), 2.22 (s, 3H, CH_3_). ^13 ^C NMR (75 MHz, DMSO-*d*_6_, ppm) *δ* 161.40, 150.29 (2 C), 150.21, 141.43, 140.72, 140.63, 136.52, 134.40, 129.13 (2 C), 126.73 (2 C), 126.37, 125.28, 124.95, 122.52, 122.19, 121.57, 120.69, 120.33, 119.68, 110.08, 109.97, 45.53, 20.58. HRMS (MALDI) calcd for C_27_H_22_N_4_O [M + H]^+^: 419.1866, found: 419.1854.

##### 2.1.8.3. (E)-N'-((9-(2-Fluorobenzyl)-9H-carbazol-3-yl)methylene)isonicotinohydrazide (12c)

Yellow solid; yield 73.8%; m.p. 220.3–221.5 °C. IR (KBr) cm^−1^: 3366, 3050 (NH), 1651 (C = O), 1589 (C = N). ^1^H NMR (300 MHz, DMSO-*d*_6_, ppm) *δ* 12.07 (s, 1H, NH), 8.82 (s, 2H, Ar-H), 8.68 (s, 1H, CH = N), 8.57 (s, 1H, pyridine–H), 8.26 (s, 1H, pyridine–H), 8.00–7.83 (m, 3H, Ar-H), 7.66 (dd, 2H, *J* = 26.4, 8.4 Hz, pyridine–H), 7.46 (t, 1H, *J* = 7.6 Hz, Ar-H), 7.35–7.16 (m, 3H, Ar-H), 7.00 (t, 1H, *J* = 7.2 Hz, Ar-H), 6.89 (t, 1H, *J* = 7.7 Hz, Ar-H), 5.74 (s, 2H, CH_2_). ^13 ^C NMR (75 MHz, DMSO-*d*_6_, ppm) *δ* 161.68, 161.43, 150.29 (2 C), 150.14, 141.38, 140.70, 140.55, 129.53 (d, 1 C, *J* = 8.1 Hz), 128.66 (d, 1 C, *J* = 4.3 Hz), 126.44, 125.48, 125.05, 124.58 (d, 1 C, *J* = 3.4 Hz), 122.59, 122.24, 121.56 (2 C), 120.71, 120.28, 119.86, 115.67, 115.39, 110.03, 109.86, 40.23. HRMS (MALDI) calcd for C_26_H_19_FN_4_O [M + H]^+^: 423.1616, found: 423.1612.

##### 2.1.8.4. (E)-N'-((9-(2,4-Dichlorobenzyl)-9H-carbazol-3-yl)methylene)isonicotinohydrazide (12d)

Yellow solid; yield 64.2%; m.p. 209.1–210.3 °C. IR (KBr) cm^−1^: 3400, 3137 (NH), 1657 (C = O), 1597 (C = N). ^1^H NMR (300 MHz, DMSO-*d*_6_, ppm) *δ* 12.04 (s, 1H, NH), 8.88–8.73 (m, 2H, Ar-H), 8.64 (s, 1H, CH = N), 8.58 (d, 1H, *J* = 1.6 Hz, pyridine–H), 8.33 (d, 1H, *J* = 7.7 Hz, pyridine–H), 7.96–7.69 (m, 4H, Ar-H and pyridine–H), 7.68–7.17 (m, 5H, Ar-H), 6.42 (d, 1H, *J* = 8.4 Hz, Ar-H), 5.76 (s, 2H, CH_2_). ^13 ^C NMR (75 MHz, DMSO-*d*_6_, ppm) *δ* 161.38, 150.28 (2 C), 150.01, 141.34, 140.67, 140.50, 133.60, 132.73, 132.67, 129.09, 128.41, 127.68, 126.60, 125.74, 125.18, 122.67, 122.31, 121.54 (2 C), 120.84, 120.34, 120.07, 109.92, 109.73, 54.91. HRMS (MALDI) calcd for C_26_H_18_Cl_2_N_4_O [M + H]^+^: 473.0930, found: 473.0941.

### 2.2. Evaluation of antibacterial activity in vitro

The *in vitro* antimicrobial and antifungal activities of the synthesised compounds were evaluated to obtain the minimum inhibitory concentrations (MICs), using a 96-well microtiter plate and a serial dilution method. Gatifloxacin and moxifloxacin were used as positive controls and DMSO as a negative control. The micro-organisms used in the present study were two Gram-positive strains (*Staphylococcus aureus* 4220, *Streptococcus mutans* 3289), one clinical isolate of multidrug-resistant Gram-positive bacterial strain (*Methicillin-resistant Staphylococcus aureus CCARM* 3167), one Gram-negative strain (*Escherichia coli* 1924) and one fungus (*Candida albicans* 7535). The bacteria were grown to mid-log phase in Mueller-Hinton broth and diluted 1000-fold in the same medium. Stock solutions of the test compounds in dimethyl sulfoxide were prepared and then poured into 96-well plates. The final concentration of 0.5–64 µg/mL underwent a twofold serial dilution^14^^,^^24^. The bacteria were suspended and contained approximately 10^5^ CFU/mL. These were applied to 96-well plates with a serial dilution and incubated at 37 °C for 24 h. The bacteria growth was measured from the turbidity at 630 nm using a microplate reader. All experiments were carried out in triplicate. Their MBC and MFC values were also determined (only for the compounds with MIC values of < 256 μg/mL).

### 2.3. MTT assay

Cell viability has been determined using the 3-(4,5-dimethylthiazol-2-y1)-2,5-diphenyltetrazolium Bromide (MTT, Sigma–Aldrich, Milan, Italy) assay. Cells have been seeded on 48 well plates and grown in complete medium. Before being treated, cells have been starved in serum-free medium for 24 h for allowing cell cycle synchronisation. 72 h after treatments, fresh MTT, re-suspended in PBS, was added to each well (final concentration (0.5 mg/mL). After 2 h incubation at 37 °C, cells have been lysed with DMSO, and then optical density was measured at 570 nm using a microplate reader. At least six doses of the studied compounds, solubilised in DMSO (0.1% final concentration), have been evaluated and each experiment has been performed in triplicate. The absorbance values have been used to determine the IC_50_ for each cell line using GraphPad Prism 5 Software (GraphPad Inc., San Diego, CA).

### 2.4. Docking study

Preliminary docking was performed to evaluate whether binding to DHFR might account for the bactericidal effect of the compounds. All docking studies were carried out using Discovery Studio 2017 (Accelrys, San Diego, CA). Docking studies were performed according to our previous report^25^. The crystal structure data were obtained from the protein data bank (*E. coli* DHFR PDB_ID: 1RX7). Enzyme structures were checked for missing atoms, bonds and contacts. Hydrogen atoms were added to the enzyme structure. Water molecules and bound ligands were manually deleted. Then the protein was refined with CHARMm. The structures of compounds were sketched in 2D and converted into 3D using the DS molecule editor. Automated docking studies were carried out to investigate the binding mode of compounds **8b** and **8f** utilizing DS-CDOCKER protocol. The pose with the top CDOCKER_INTERACTION_ENERGY was chosen for analysing the binding features of compounds **8b** and **8f** with DHFR.

### 2.5. Inhibition of DHFR activities in vitro

The inhibition of DHFR activities of compound **8f** was measured using ELISA kits (Mlbio, Shanghai, China) under different concentrations (0, 0.1, 0.3, 1, 3, 10 µmol/L), according to the manufacturer’s instructions.

## 3. Results and discussion

### 3.1. Chemistry

**Scheme 1.**
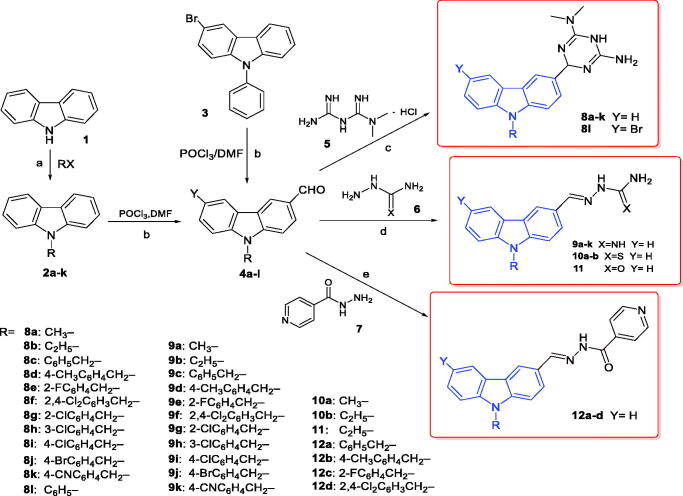
Synthetic scheme for the synthesis of the target compounds. Reagents and conditions: (a) DMC, DABCO, 95 °C, 24 h (**2a**); KOH, 25 °C, 2 h (**2b**); NaH, DMF, 25 °C, 16 h (**2c–k**) (b) POCl_3_, DMF, 90 °C, 8–18 h (c) AcOH, 120 °C, 4–8 h (d) CH_2_CH_3_OH, HCl, 40 °C, 6 h (**9a–k**); CH_3_OH, AcOH, 68 °C, 4 h (**10a–b**,**11**) (e) CH_2_CH_3_OH, AcOH, 70 °C, 5 h.

The synthetic routes used for the construction of target compounds **8a–l**, **9a–k**, **10a–b**, **11** and **12a–d** are outlined in Scheme 1. A series of intermediates (2) was obtained from carbazole (1) via N-alkylation or N-arylation. Next, we performed a formylation reaction (Vilsmayer-Hack) on the intermediates (2) and commercially available compound **3** in the presence of POCl_3_ and DMF to obtain 9-substituted carbazole-3-carbaldehyde analogues **4** in good yields. Compounds **8a–l** were prepared by the reaction of each of the series **4** compounds with metformin hydrochloride in acetic acid. Compounds **9a–k** were synthesised via the reactions of each of the series **4** compounds with aminoguanidine hydrochloride in ethanol with a catalytic amount of concentrated hydrochloric acid. Compounds **10a–b** and **11** were prepared by reacting compounds **4** with thiosemicarbazide and semicarbazide, respectively, in the presence of acetic acid in methanol under reflux. Next, we reacted compounds **4** with isonicotinic in the presence of acetic acid in alcohol to obtain compounds **12a–d**.

### 3.2. Evaluation of in vitro antimicrobial activities

The antimicrobial activities of the synthesised compounds were then tested against a series of pathogenic bacterial and fungal strains. Several different bacterial strains and one fungal strain were susceptible to most of the compounds tested with MICs in the range of 1–64 µg/ml. N-aryl carbazole derivatives containing an aminoguanidine or 1,4-dihydro-1,3,5-triazine moiety (**8c–l** and **9c–k**) exhibited potent antibacterial activities with MICs ranging from 0.5 to 16 µg/ml. These compounds also exhibited antifungal activity against *Candida albicans* 7535 with MICs ranging from 1 to 32 µg/ml.

Compounds **8f**, **8l**, **9d** and **9e** exhibited strong antibacterial activity against two Gram-positive strains (*methicillin-resistant Staphylococcus aureus* (MRSA CCARM 3167) and *Staphylococcus aureus* RN4220) and one Gram-negative strain (*Escherichia coli* 1924) with MIC values of 0.5 or 1 µg/ml. Furthermore, the antibacterial activities of compound **8f** against Gram-positive bacterial strains (*S. aureus* RN4220 and *Streptococcus mutans* 3289) were comparable to the positive control drugs gatifloxacin and moxifloxacin. In addition, compound **8f** displayed considerable potency against MRSA CCARM 3167 (MIC of 0.5 μg/ml) that was two- and four-fold greater compared with moxifloxacin (MIC of 1 μg/ml) and gatifloxacin (MIC of 2 μg/ml) respectively. In addition, compound **8f** demonstrated a strong inhibitory activity (MIC of 0.5 μg/ml) against *E. coli* 1924, which was four-fold greater than the activities of moxifloxacin (MIC of 2 μg/ml) and gatifloxacin (MIC of 2 μg/ml), respectively. In contrast, compound **8f** showed a weaker activity against the fungus *C. albicans* 7535 compared with the positive controls.

Moreover, we determined the minimum bactericidal concentrations (MBCs) and minimum fungicidal concentrations (MFCs) for compounds whose MIC value less than 256 µg/ml against strains. MBCs and MFCs are defined as the lowest bactericidal or fungicidal concentration required to kill a particular bacterial strain or the fungal strain over a fixed incubation time. Most of the series **8** compounds displayed MBCs at 1–2 fold higher than their MICs against the strains tested. Compounds **9i** and **9k** also had four-fold higher MIC values compared with their MBCs against *S. aureus* RN4220 and MRSA CCARM 3167, respectively. The MBC/MIC ratio of compounds **9 g** and **9j** were ≥4 for *S. mutans* 3289. Compounds **9c**, **9e**, **9h** and **9i** demonstrated MFC values that were four-fold higher than their MICs against *Candida albicans* 7535. Notably, bacteria appeared to be less tolerant to the series **8** compounds compared with series **9** compounds. When we altered the phenyl ring substituents of compounds **8a–l**, we observed a significant effect on the potency of their antimicrobial activities. The order of antibacterial activities were as follows: phenyl group > 2,4-dichloro-substitutions > 4-CH_3_(for compounds bearing an electron-donating group) > halogen substitutions > benzyl group > 4-CN (for compounds bearing an electron-withdrawing group) > alkyl group. Furthermore, bromo- and chloro-substitutions on the phenyl ring in compounds **8a–l** were observed to improve their antifungal activity against *C. albicans* 7535. The antibacterial activity order of compounds **9a–k** was similar to compounds **8a–l**. Compounds in series **10a–b**, **11** and **12a–d** generally showed weak activity against all the strains tested in this study, with MICs greater than 256 µg/ml. As shown in Table 1, most of the compounds exhibited equivalent or higher potency (with MIC values in the range of 0.5 to 16 µg/ml) towards MRSA CCARM 3167 compared with the positive controls gatifloxacin and moxifloxacin (MICs of 2 and 1 µg/ml, respectively).

**Table 1.** Inhibitory activities (MIC/MBC or MFC, μg/mL) of compounds **8a–l**, **9a–k**, **10a–b**, **11** and **12a–d** against various bacteria and fungi.

Compd	R			Gram-positive strains			Gram-negative strain	Fungus
				*S. aureus*	MRSA CCARM	*S. mutans*	*E. coli*	*C. albicans*
	Y	R	X	4220^a^	3167^b^	3289^c^	1924^d^	7535^e^
**8a**	H	CH_3_	—	16/16	16/16	64/64	8/8	128/128
**8b**	H	C_2_H_5_	—	64/64	8/8	32/128	8/16	64/128
**8c**	H	benzyl	—	4/4	0.5/1	2/4	0.5/1	32/32
**8d**	H	4-CH_3_C_6_H_4_CH_2_-	—	1/1	0.5/0.5	2/2	0.5/0.5	4/8
**8e**	H	2-FC_6_H_4_CH_2_-	—	4/4	0.5/0.5	0.5/4	0.5/1	32/32
**8f**	H	2,4-2ClC_6_H_3_CH_2_-	—	0.5/0.5	0.5/0.5	0.5/1	0.5/0.5	2/2
**8g**	H	2-ClC_6_H_4_CH_2_-	—	2/2	0.5/1	2/4	1/1	1/1
**8h**	H	3-ClC_6_H_4_CH_2_-	—	4/4	0.5/1	2/8	1/1	2/2
**8i**	H	4-ClC_6_H_4_CH_2_-	—	2/2	0.5/0.5	2/2	1/1	1/1
**8j**	H	4-BrC_6_H_4_CH_2_-	—	2/2	0.5/1	2/2	1/1	2/2
**8k**	H	4-CNC_6_H_4_CH_2_-	—	16/16	16/32	16/16	8/8	8/8
**8l**	Br	C_6_H_5_-	—	1/1	0.5/0.5	1/2	0.5/1	1/1
**9a**	—	CH_3_	NH	2/4	2/2	4/4	2/2	4/8
**9b**	—	C_2_H_5_	NH	4/4	8/8	8/16	4/4	4/8
**9c**	—	benzyl	NH	2/2	1/2	1/2	1/2	1/4
**9d**	—	4-CH_3_C_6_H_4_CH_2_-	NH	1/2	0.5/0.5	0.5/1	0.5/1	1/2
**9e**	—	2-FC_6_H_4_CH_2_-	NH	2/4	0.5/0.5	1/1	1/1	1/4
**9f**	—	2,4-2ClC_6_H_3_CH_2_-	NH	2/4	1/2	2/2	2/2	16/16
**9g**	—	2-ClC_6_H_4_CH_2_-	NH	1/2	1/1	1/4	1/1	2/4
**9h**	—	3-ClC_6_H_4_CH_2_-	NH	1/2	0.5/0.5	1/2	2/2	2/8
**9i**	—	4-ClC_6_H_4_CH_2_-	NH	1/4	0.5/0.5	1/1	2/2	2/8
**9j**	—	4-BrC_6_H_4_CH_2_-	NH	2/2	0.5/1	2/16	2/2	4/8
**9k**	—	4-CNC_6_H_4_CH_2_-	NH	2/4	2/8	2/4	2/2	2/4
**10a**	—	CH_3_	S	256/256	>256	>256	>256	>256
**10b**	—	C_2_H_5_	S	>256	>256	>256	>256	>256
**11**	—	C_2_H_5_	O	>256	>256	>256	>256	>256
**12a**	—	benzyl	—	>256	>256	>256	>256	>256
**12b**	—	4-CH_3_C_6_H_4_CH_2_-	—	>256	>256	>256	>256	>256
**12c**	—	2-FC_6_H_4_CH_2_-	—	>256	>256	>256	>256	>256
**12d**	—	2,4-2ClC_6_H_3_CH_2_-	—	>256	>256	>256	>256	>256
Gatifloxacin				0.25/0.25	2/2	0.25/n.t.^f^	2/2	0.5/0.5
Moxifloxacin				0.25/0.25	1/2	0.25/n.t.	2/2	0.5/0.5

CCARM: Culture Collection Antimicrobial Resistant Microbes; KCTC: Korean Collection for Type Cultures.

^a^*Staphylococcus aureus RN* 4220.

^b^*Methicillin-resistant S. aureus CCARM* 3167.

^c^*Streptococcus mutans 3289.* ^d^*Escherichia coli KCTC* 1924. ^e^*Candida albicans* 7535.

^f^n.t.: Not test.

To summarise, our findings revealed that the compounds in series **8** exhibited significantly higher antimicrobial activities compared with the compounds in the other four series. This suggests that the presence of a dihydrotriazine moiety is critical to the potency of these carbazole derivatives. These results therefore provide further evidence to suggest that aryl groups and the dihydrotriazine moiety play critical roles in the activity of these carbazole compounds.

### 3.3 Cell viability

To determine whether the synthesised compounds were selectively toxic towards microbes and not human cells, we evaluated the cytotoxicity of six compounds in two gastric cancer cell lines (SGC-7901 and AGS) and a normal human liver cell line (L-02) using a standard technique. As shown in Table 2, the cytotoxicities of compounds **8b**, **8d**, **8f** and **8k** were lower than that of compounds **9b** and **9e**. Compounds **8b**, **8d**, **8f** and **8k** had no effect on human liver cell viability at their MICs and, in addition, their IC_50_ values were significantly higher than their MIC values. However, the IC_50_ values of the series **9** compounds were close to their MIC values, which make it likely that the promising antibacterial activity of these compounds is due to their cytotoxic properties.

**Table 2.** Cytotoxic activity (IC_50_^a^, µg/mL) of compounds **8b**, **8d**, **8f**, **8k**, **9b** and **9e** against human cell lines.

Compd	*In vitro* cytotoxicity IC_50_^a^ (μg/mL)		
	SGC-7901^b^	AGS^b^	L-02^c^
**8b**	>33.4	>33.4	>33.4
**8d**	7.1	9.9	10.1
**8f**	10.0	7.8	9.4
**8k**	>42.1	>42.1	>42.1
**9b**	1.8	1.2	3.8
**9e**	1.5	0.4	1.1

^a^IC_50_ is the concentration required to inhibit the cell growth by 50%. Data represent the average of three independent experiments running in triplicate. Variation was generally between 5 and 10%.

^b^Human gastric cancer cells.

^c^Human normal hepatic cells.

Compounds in series **8a–l**, containing a dihydro-1,3,5-triazine ring moiety, were generally found to have more potent antimicrobial effects but less cytotoxicity compared with the corresponding series **9a–l** compounds that comprised an aminoguanidine moiety. Interestingly, our results also showed that compound **9 b** was selectively more toxic to human cancer cells compared with normal human cells. The IC_50_ value is about three times higher in normal cells than AGS cell lines. Further studies are required to investigate if this compound has any potential as a new anticancer agent.

### 3.4. Docking analysis

**Figure 2.**
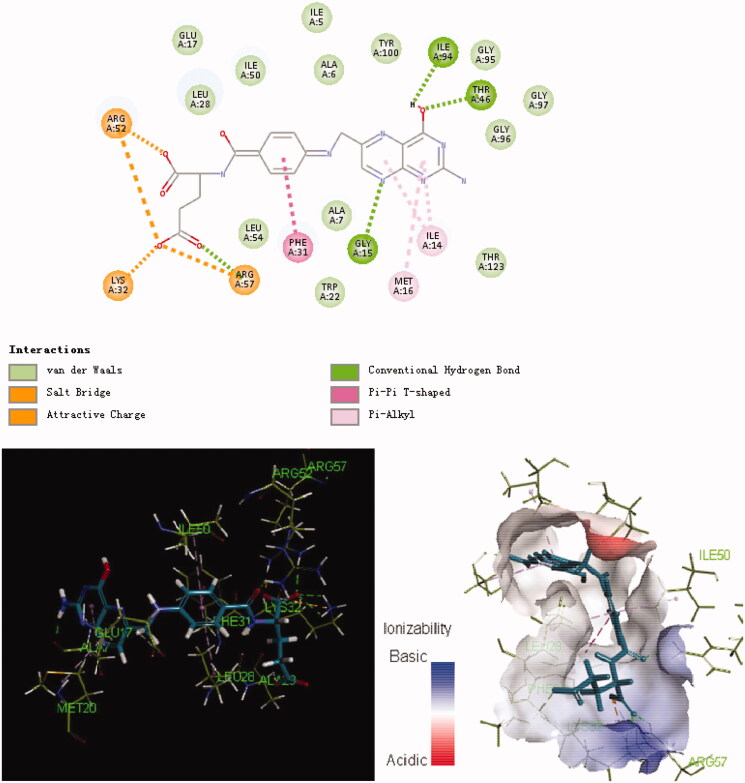
Binding mode of folate inside the *E. coli* DHFR active site.

**Figure 3.**
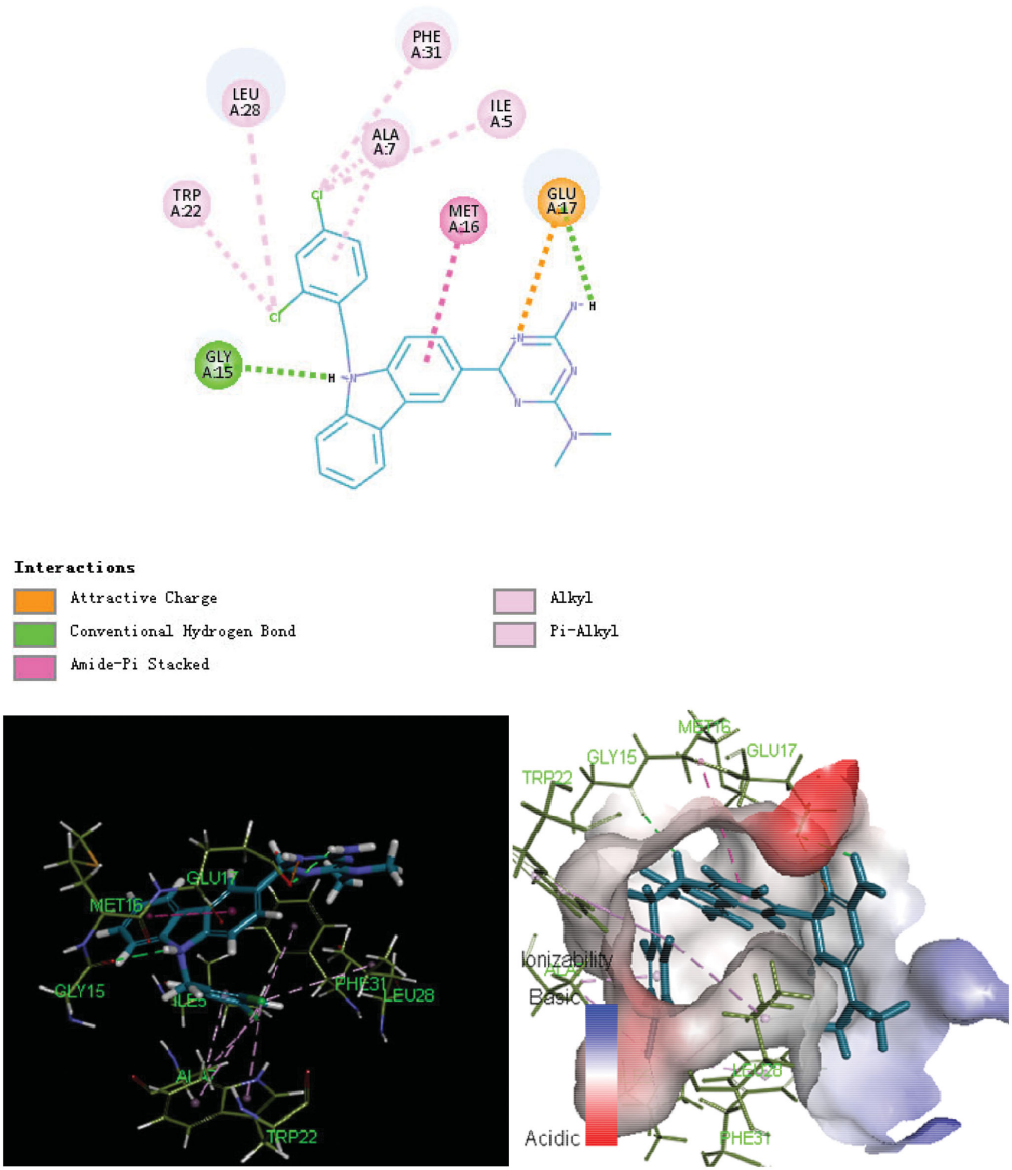
Binding mode of **8f** inside the *E. coli* DHFR active site.

**Figure 4.**
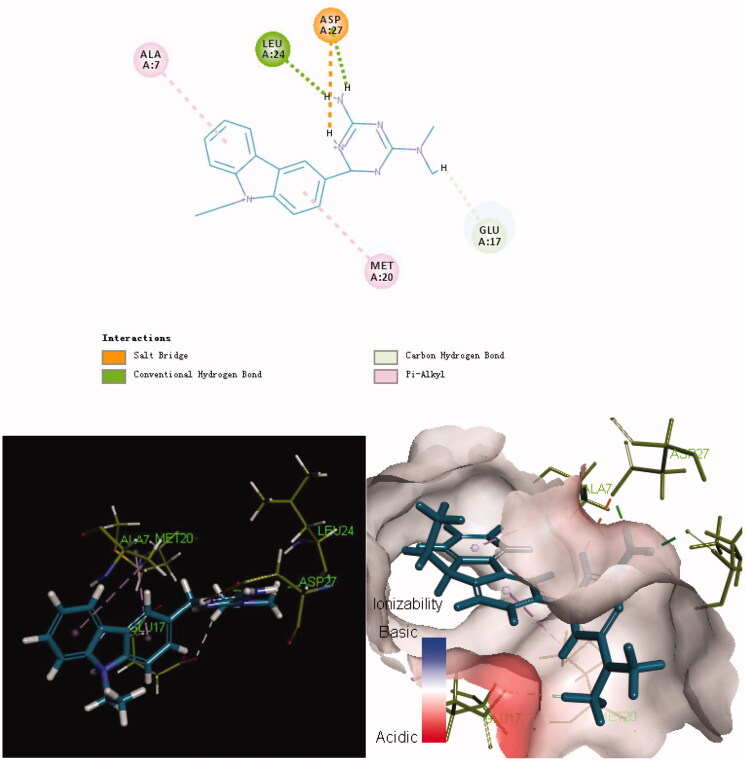
Binding mode of **8b** inside the *E. coli* DHFR active site.

The potent and selective antimicrobial activities of the series **8** compounds prompted us to study the binding of these derivatives to their potential target, *E. coli* DHFR. In 3D binding mode, alkyl chains (ethyl and methylene) at the 9-position of carbazole possess considerable flexibility (Figures 2–4). As shown in Figure 3, the aryl group at the 9-position of carbazole in compound **8f** inserted deeply into the active pocket of DHFR (composed of Ala7, Trp22, Leu28 and Phe31). Moreover, both the folic acid substrate and compound **8f** have a nitrogen atom in their heterocyclic rings that forms a hydrogen bond with the *E. coli* DHFR residue Gly15. The hydrophobic interactions between compound **8f** and residues in the DHFR active pocket were enhanced due to the presence of the carbazole moiety. In addition, the primary amine group at the N-3 position of the carbazole ring plays an important role in binding to the active site of *E. coli* DHFR. A salt bridge was formed between the primary amine and Glu17. To summarise, docking results suggested that **8f**, the compound with the most therapeutic potential, has interacted with the critical active-site residues of *E. coli* DHFR.

### 3.5. Inhibition studies of compound 8f with DHFR

**Figure 5.**
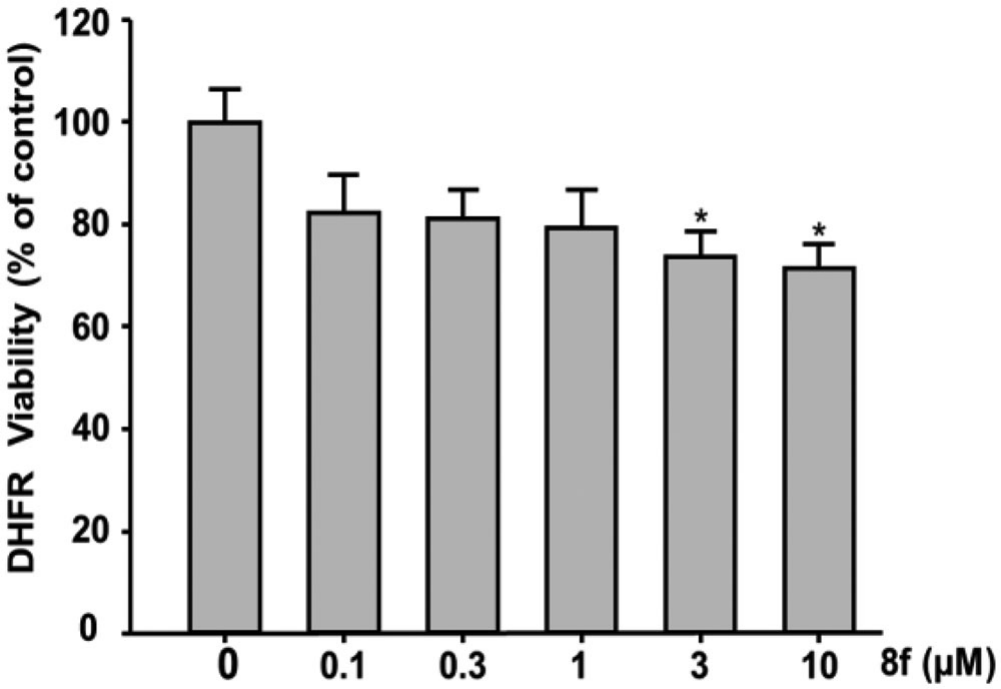
Inhibition of DHFR of compound **8f**.

To investigate whether compound **8f** can bind to and block the active site of DHFR, we performed *in vitro* enzyme assays to test the inhibitory effect of compound **8f** (MIC of 0.5 μg/ml) on DHFR activity (Figure 5). At concentrations of 3 and 10 μmol/L, compound **8f** decreased DHFR activity by 71% compared with the negative control. The results indicated that compound **8f** exerts its antibacterial activity via binding to DHFR, which however might not be the only mechanism of action.

## 4. Conclusions

We have designed, synthesised and evaluated the antibacterial and antifungal activities of five series of novel carbazole derivatives. Compound **8f** showed the most potential as a therapeutic agent, with an MIC of 0.5–2 µg/ml against selected bacterial strains. Furthermore, compound **8f** also was the least cytotoxic to SGC-7901, AGS and L-02 cells. Compound **9d** also exhibited strong antibacterial activity with the cancer therapeutic potential. Therefore, the clinical potential of carbazole derivatives **8f** and **9d** could be explored further for future applications in antitumour and antimicrobial therapies.

Docking simulation and *in vitro* enzyme activity assays suggested that binding to DHFR might account for the antimicrobial activity of the compound**s**. Further studies of the mechanisms of action of these compounds are currently underway in our laboratories and will be reported in due course.
